# Entangled States are Typically Incomparable

**DOI:** 10.1007/s00220-026-05711-4

**Published:** 2026-08-28

**Authors:** Vishesh Jain, Matthew Kwan, Marcus Michelen

**Affiliations:** 1 University of Illinois Chicago, Chicago, USA; 2 Institute of Science and Technology Austria (ISTA), Klosterneuburg, Austria; 3 Northwestern University, Evanston, USA

## Abstract

Consider a bipartite quantum system, where Alice and Bob jointly possess a pure state $|\psi \rangle$. Using local quantum operations on their respective subsystems, and unlimited classical communication, Alice and Bob may be able to transform $|\psi \rangle$ into another state $|\phi \rangle$. Famously, *Nielsen’s theorem* [28] provides a necessary and sufficient algebraic criterion for such a transformation to be possible (namely, the *entanglement spectrum* of $|\phi \rangle$ should *majorise* the entanglement spectrum of $|\psi \rangle$). In the paper where Nielsen proved this theorem, he conjectured that in the limit of large dimensionality, for *almost all* pairs of states $|\psi \rangle , |\phi \rangle$ (according to the natural unitary invariant measure) such a transformation is not possible. That is to say, typical pairs of quantum states $|\psi \rangle , |\phi \rangle$ are entangled in fundamentally different ways, that cannot be converted to each other via local operations and classical communication. Via Nielsen’s theorem, this conjecture can be equivalently stated as a conjecture about majorisation of spectra of random matrices from the so-called *trace-normalised complex Wishart–Laguerre ensemble*. Concretely, let *X* and *Y* be independent n×m random matrices whose entries are i.i.d. standard complex Gaussians; then Nielsen’s conjecture says that the probability that the spectrum of $X X^\dagger / \operatorname {tr}(X X^\dagger )$ majorises the spectrum of $Y Y^\dagger / \operatorname {tr}(Y Y^\dagger )$ tends to zero as both *n* and *m* grow large. We prove this conjecture, and we also confirm some related predictions of Cunden et al. [12].

## Introduction

Suppose a bipartite pure quantum state is distributed to two parties Alice and Bob. Imagine that Alice and Bob then travel to distant labs; after this point, they may only perform quantum operations on their own subsystem, though they are free to coordinate their operations via classical communication (e.g., they can share the results of any measurements). This paradigm is known as LOCC (local operations with classical communication), and plays a fundamental role in quantum information theory. See the Nielsen–Chuang monograph [29] for a general introduction to quantum information theory, and see the survey of Chitambar, Leung, Mančinska, Ozols, and Winter [10] for a thorough introduction to the LOCC paradigm.

The LOCC paradigm naturally defines a partial order (called *LOCC-convertibility* or *entanglement transformation*) on the set of bipartite pure states $\mathbb C^{n}\otimes \mathbb C^{m}$ for any dimensions $n,m\in \mathbb N$. Namely, for a pair of states $|\psi \rangle ,|\phi \rangle \in \mathbb C^{n}\otimes \mathbb C^{m}$, we write $|\psi \rangle \rightarrow |\phi \rangle$ (read “$|\psi \rangle$ transforms to $|\phi \rangle$”) if and only if, starting from the state $|\psi \rangle$, it is possible for Alice and Bob to coordinate some sequence of local operations to guarantee that they end up with the state $|\phi \rangle$.

In 1999, Nielsen [28] found a beautiful connection between LOCC-convertibility and the mathematical theory of *majorisation*. For the precise statement, and more context, the reader should refer to [28] (and Chapter 12.5.1 of Nielsen–Chuang [29]), but as a brief summary: although Alice and Bob share a pure state $|\psi \rangle$, Alice can interpret her subsystem as a *mixed* state (a statistical ensemble of pure states in $\mathbb C^n$). This mixed state can be described by a *density operator* $\rho _\psi =\operatorname {tr}_2(|\psi \rangle \langle \psi |)$ (a Hermitian linear operator $\mathbb C^n\rightarrow \mathbb C^n$ obtained via a *partial trace*, “tracing out” Bob’s subsystem). The eigenvalues of $\rho _\psi$ are all nonnegative real numbers, summing to 1^1^. Nielsen’s theorem is that LOCC-convertibility $|\psi \rangle \rightarrow |\phi \rangle$ is equivalent to the property that the spectrum of $\rho _\phi$ *majorises* the spectrum of $\rho _\psi$: the sum of the *k* smallest^2^ eigenvalues of $\rho _\psi$ should be at most the sum of the *k* smallest eigenvalues of $\rho _\phi$, for all k≤n.

Nielsen’s theorem has a number of consequences. In particular, it lays bare the fact that (if n,m≥3) there are fundamentally different types of entanglement that cannot be LOCC-converted to each other: there exists a pair of states $|\psi \rangle ,|\phi \rangle \in \mathbb C^{n}\otimes \mathbb C^{m}$ such that $|\psi \rangle \not \rightarrow |\phi \rangle$ and $|\phi \rangle \not \rightarrow |\psi \rangle$. Nielsen conjectured that this phenomenon is actually *typical*: if $|\psi \rangle , |\phi \rangle$ are independent random states (both sampled from the Haar measure, with respect to unitary transformations, on the unit sphere in $\mathbb C^{n}\otimes \mathbb C^{m}$), then the probability of the event $|\psi \rangle \rightarrow |\phi \rangle$ tends to zero as $n,m\rightarrow \infty$. This conjecture has remained unproved for the last 25 years, though it is supported by strong numerical evidence (cf. the very convincing computations of Cunden et al. [12]). There are also quite convincing heuristic arguments for why it should be true: a brief heuristic argument was given by Nielsen [28], and another heuristic argument via integral geometry was given by Życzkowski and Bengtsson [42] (see also their monograph [7] on the geometry of entanglement). Also, an *infinite-dimensional* counterpart to Nielsen’s conjecture (with a *topological* notion of “typical”) was proved by Clifton, Hepburn and Wüthrich [11]. We note that Nielsen’s conjecture does not currently appear to have a direct application to protocols or operational tasks; it should be viewed as a mathematical conjecture about the structure of entanglement transformation.

The main purpose of this paper is to prove Nielsen’s conjecture. Due to Nielsen’s characterisation of LOCC-convertibility in terms of majorisation, we can actually completely abandon the language of quantum information theory: for a random pure state $|\psi \rangle \in \mathbb C^{n}\otimes \mathbb C^{m}$, the density operator $\rho _{\psi }$ can be interpreted^3^ as a random matrix sampled from the *trace-normalised complex Wishart–Laguerre ensemble*, defined as follows.

### Definition

For parameters $n,m\in \mathbb N$, the *complex Wishart–Laguerre ensemble* is the distribution of the random Hermitian matrix $GG^\dagger$, where “†” denotes Hermitian transpose, and $G\in \mathbb C^{n\times m}$ is an n×m matrix with independent complex standard Gaussian entries^4^. Moreover, by the *trace-normalised complex Wishart–Laguerre ensemble*, we mean the distribution of $M/\operatorname {tr}(M)$, where *M* is sampled from the complex Wishart–Laguerre ensemble.

So, Nielsen’s conjecture is equivalent to the following theorem, which is the main result of this paper.

### Theorem 1.1

Let $A^{(1)},A^{(2)}\in \mathbb C^{n\times n}$ be independent samples from the trace-normalised complex Wishart–Laguerre ensemble with parameters *n*, *m*. Let $\lambda _1^{(1)}\ge \dots \ge \lambda _n^{(1)} \ge 0$ and $\lambda _1^{(2)}\ge \dots \ge \lambda _n^{(2)} \ge 0$ be the eigenvalues of $A^{(1)}$ and $A^{(2)}$, respectively. Then

$$\begin{aligned} \mathbb P\big [\lambda _k^{(1)}+\dots +\lambda _n^{(1)}\le \lambda _k^{(2)}+\dots +\lambda _n^{(2)}\text { for all }k\le n\big ]\rightarrow 0 \end{aligned}$$

as $$n,m\rightarrow \infty $$.

We discuss the proof approach for Theorem 1.1 in a bit more detail later in this introduction (Sect. 1.3), but to give a very brief flavour: due to the so-called *eigenvalue repulsion* phenomenon, the (complex) Wishart–Laguerre spectrum has only tiny fluctuations, and existing technology in random matrix theory is not precise enough to describe the distributions of quantities of the form $\lambda _k+\dots +\lambda _n$. So, instead of considering such quantities directly, we consider a sequence of convex test functions which emphasise different parts of the spectrum (increasingly “focusing in” on the “edges” of the spectrum); this defines a sequence of linear statistics with nearly independent fluctuations, for which we can derive a multivariate central limit theorem. We can then take advantage of the Hardy–Littlewood–Pólya theorem relating convexity and majorisation, together with some analytic and combinatorial estimates.

### Remark

Our proof approach, as written, is not quantitative; we prove that the desired majorisation probability converges to zero without proving any explicit bounds on this probability. In principle, it may be possible to establish quantitative central limit theorems for the complex Wishart–Laguerre spectrum, which would provide an explicit bound, though it seems that substantial new ideas would be required to precisely characterise the asymptotic rate of decay of the majorisation probability (computer experiments by Cunden, Facchi, Florio and Gramegna [12] indicate that the probability has order of magnitude $n^{-\theta }$ for some constant θ>0, assuming that the ratio *m*/*n* is held constant).

### Remark

We have only discussed LOCC-convertibility of *bipartite* quantum states, and it is natural to also ask about *multipartite* states with more than two subsystems. The theory of LOCC-convertibility becomes much more challenging in this general setting (in the tripartite case, a necessary and sufficient condition is known [37], but it is rather complicated). However, our proof of Nielsen’s conjecture automatically implies a similar result for any number of subsystems (e.g., if there are three subsystems, we can simply view two of the subsystems as comprising a single larger subsystem to reduce to the bipartite case; this only makes LOCC-convertibility more permissive). Actually, for more than two subsystems one expects much stronger results along these lines; see [18, 34].

### Remark

It would be interesting to investigate whether similar ideas can be extended to other natural random measures and related partial orders. For fixed-trace orthogonal or symplectic Wishart ensembles, one would need analogues of the multivariate linear-statistics central limit theorems used in Theorem 2.3, 2.7, together with sufficiently precise control of the corresponding covariance structure after trace-normalisation. For more general measures on the simplex, such as Dirichlet-type laws, one obstacle is that the convenient exact order-statistic representation used in section 3 is no longer available in the same form. One could also ask for analogues in thermomajorisation, but there the relevant order is governed by thermo-Lorenz curves rather than ordinary majorisation, so the Hardy–Littlewood–Pólya convex-function reduction used here is not directly available.

In addition to Theorem 1.1, we also prove some other related results, which we describe next.

### The uniform measure on the simplex

For any possible outcome *M* of the trace-normalised complex Wishart–Laguerre ensemble, the eigenvalues are nonnegative real numbers that sum to 1. That is to say, the spectrum can be interpreted as a point in the (n-1)-simplex

$$\begin{aligned} \Delta _{n-1}=\{{x}\in [0,\infty )^{n}:x_{1}+\dots +x_{n}=1\}, \end{aligned}$$

and Nielsen’s conjecture can be interpreted as a conjecture about majorisation of two independent random points sampled from a particular probability distribution (defined in random-matrix-theoretic terms) on $\Delta _{n-1}$.

Of course, there are other interesting distributions on $\Delta _{n-1}$; perhaps the most natural (and most tractable) such measure is the *uniform* measure on $\Delta _{n-1}$. Cunden, Facchi, Florio and Gramegna [13] proved the natural analogue of Nielsen’s conjecture for this measure. Their proof was fundamentally non-quantitative (it critically used Kolmogorov’s zero-one law), but based on numerical experiments and heuristic comparison with persistence probabilities of random walks, they predicted that the majorisation probability should decay “polynomially fast” (specifically, they predicted that the majorisation probability should scale like $n^{-\theta }$ for some positive constant $\theta \approx 0.41$). We are able to prove a bound of this shape (though presumably not with an optimal value of θ), as follows.

#### Theorem 1.2

Let $(X_k^{(1)})_{k=1}^n,(X_k^{(2)})_{k=1}^n$ be two independent uniform random vectors in $\Delta _{n-1}$, and denote by $(\lambda _k^{(1)})_{k=1}^n,(\lambda _k^{(2)})_{k=1}^n$ their *decreasing rearrangements* (i.e., the results of sorting $(X_k^{(1)})_{k=1}^n,(X_k^{(2)})_{k=1}^n$ in decreasing order). Then

$$\begin{aligned} \mathbb P\big [\lambda _k^{(1)}+\dots +\lambda _n^{(1)}\le \lambda _k^{(2)}+\dots +\lambda _n^{(2)}\text { for all }k\le n\big ]= O((n/\log n)^{-1/16}). \end{aligned}$$

As observed by Cunden, Facchi, Florio and Gramegna [13], this result actually has a quantum-information-theoretic interpretation: in the same way that Nielsen’s conjecture studies the resource of *entanglement* (i.e., the operations that can be made without introducing new entanglement into the system), Theorem 1.2 can be interpreted as a theorem about the resource of *coherence*. We direct the reader to [13], and the references therein, for more details.

### Approximate LOCC-convertibility

So far, we have only been concerned about *exact* LOCC-convertibility: we write $|\psi \rangle \rightarrow |\phi \rangle$ if it is possible for Alice and Bob to orchestrate a sequence of local quantum operations (with unlimited classical communication) which *guarantee*, with probability 1, that $|\psi \rangle$ will be converted to $|\phi \rangle$. It is natural to ask how the situation changes if we allow some probability of failure.

Shortly after Nielsen’s 1999 paper, Vidal [40] found a generalisation of Nielsen’s theorem along these lines. For bipartite states $|\psi \rangle ,|\phi \rangle \in \mathbb C^{n}\otimes \mathbb C^{m}$, he proved that the maximum possible success probability $\Pi (|\psi \rangle \rightarrow |\phi \rangle )$ for the task of converting $|\psi \rangle$ to $|\phi \rangle$ (in the LOCC paradigm) is exactly

$$\begin{aligned} \min _{1\le k\le n} \frac{\lambda _k^{(1)}+\lambda _{k+1}^{(1)}+\dots +\lambda _n^{(1)}}{\lambda _k^{(2)}+\lambda _{k+1}^{(2)}+\dots +\lambda _n^{(2)}}, \end{aligned}$$

where $\lambda _1^{(1)}\ge \dots \ge \lambda _n^{(1)}$ are the eigenvalues of $\rho _{\psi }$ and $\lambda _1^{(2)}\ge \dots \ge \lambda _n^{(2)}$ are the eigenvalues of $\rho _{\phi }$.

In [12], Cunden, Facchi, Florio and Gramegna considered $\Pi (|\psi \rangle \rightarrow |\phi \rangle )$ for *random* states $|\psi \rangle ,|\phi \rangle \in \mathbb C^{n}\otimes \mathbb C^{m}$: with what probability can one successfully convert between two randomly entangled states? In their computer simulations, they found that the answer seems to heavily depend (in a somewhat surprising way) on the relationship between *n* and *m*. Indeed, suppose $n,m\rightarrow \infty$ with m/n=c (for some constant $c\in (0,\infty )$). If c=1, computational evidence suggests that $\Pi (|\psi \rangle \rightarrow |\phi \rangle )$ has a nontrivial limiting distribution supported on the whole interval [0, 1] (with mean about 0.58). On the other hand, if c≠1 then computational evidence suggests that $\Pi (|\psi \rangle \rightarrow |\phi \rangle )$ converges to 1 in probability.

As our final result in this paper, we confirm the latter prediction.

#### Theorem 1.3

Let $A^{(1)},A^{(2)}\in \mathbb C^{n\times n}$ be independent samples from the trace-normalised complex Wishart–Laguerre ensemble with parameters *n*, *m*. Let $\lambda _1^{(1)}\ge \dots \ge \lambda _n^{(1)}$ and $\lambda _1^{(2)}\ge \dots \ge \lambda _n^{(2)}$ denote the respective eigenvalues, and let

$$\begin{aligned} \Pi =\min _{1\le k\le n} \frac{\lambda _k^{(1)}+\lambda _{k+1}^{(1)}+\dots +\lambda _n^{(1)}}{\lambda _k^{(2)}+\lambda _{k+1}^{(2)}+\dots +\lambda _n^{(2)}}. \end{aligned}$$

Then, for any fixed ε>0 and c≠1, we have

$$\begin{aligned} \mathbb {P}[\Pi < 1 - \varepsilon ] \rightarrow 0 \end{aligned}$$

as $$n,m\rightarrow \infty $$ with m/n→c.

#### Remark 1.4

The conclusion of Theorem 1.3 cannot hold without some separation between *m* and *n*. Indeed, in the case m=n, Edelman [16] showed that the smallest eigenvalue $\lambda _{n}$ of a matrix drawn from the complex Wishart–Laguerre ensemble has probability density function given by $f(\lambda ) = ne^{-\lambda n/2}/2$. Using the concentration of the trace (as in the proof of Theorem 1.3) along with the observation that $\Pi \le \lambda _n^{(1)}/\lambda _{n}^{(2)}$, we see that for every ε<1, there exists $c_{\varepsilon } > 0$ such that $\mathbb {P}[\Pi < 1-\varepsilon ] > c_{\varepsilon }$.

Our proof of Theorem 1.3 really shows that $\mathbb {P}[\Pi < 1 - \varepsilon ] \rightarrow 0$ as long as |m-n| grows faster than $\sqrt{n \log n}$ (see Theorem 4.1 for a precise statement), and it is conceivable that existing results in random matrix theory can be used to extend the above argument (showing $\mathbb {P}[\Pi < 1-\varepsilon ] > c_{\varepsilon }$) to the range $|m - n| = O(\sqrt{n})$. However, this would still leave a “gap” of order $\sqrt{\log n}$. It would be interesting to investigate this further.

### Discussion of proof ideas

If $(\lambda _k^{(1)})_{k=1}^n$ and $(\lambda _k^{(2)})_{k=1}^n$ are the decreasing rearrangements of two independent random vectors on the simplex, sampled according to any continuous distribution, then for every *individual* *k*, it is easy to see by symmetry considerations that $\lambda _k^{(1)}+\dots +\lambda _n^{(1)}\le \lambda _k^{(2)}+\dots +\lambda _n^{(2)}$ with probability exactly 1/2. So, a first instinct for how to study majorisation of $(\lambda _k^{(1)})_{k=1}^n$ and $(\lambda _k^{(2)})_{k=1}^n$ (e.g., in the settings of Theorem 1.1, 1.2) is to study the dependence between the events that $\lambda _k^{(1)}+\dots +\lambda _{n}^{(1)}\le \lambda _k^{(2)}+\dots +\lambda _{n}^{(2)}$ for different *k*. For example, if we consider a sequence of indices $k_1<\dots <k_\ell$ which grow very rapidly or are in some other sense “well-spaced” from each other, one might hope that the corresponding events are approximately independent from each other, meaning that one might hope for an upper bound of about $2^{-\ell }$ on the majorisation probability. This type of approach has been very successful for some similar problems about majorisation in random settings (see for example [25, 30, 31] for its use in the study of random integer partitions).

This is the approach we take in our proof of Theorem 1.2. Indeed, we employ an *exact description* of the joint distribution of $(\lambda _k)_{k=1}^n$, in terms of independent exponential random variables. Via some simple approximation arguments, we can relate the quantities $\lambda _1^{(1)}+\dots +\lambda _{k}^{(1)}$ to so-called *iterated partial sums* (also called *integrated random walks*). There is a fairly extensive literature on persistence of iterated partial sums (see for example [3, 14, 36, 41]); in particular we take advantage of a general estimate due to Dembo, Ding and Gao [14].

Unfortunately, the above strategy seems to be extremely difficult to apply in the setting of Theorem 1.1. In this setting there does not seem to be any convenient exact description of the distribution of $\lambda _k+\dots +\lambda _n$, so it seems we are forced to turn to limit theorems. There is a huge body of research on central limit theorems for certain statistics of Wishart–Laguerre ensembles (see for example [1, 2, 4, 6, 9, 17, 19, 23, 35]), but none of this applies to quantities of the form $\lambda _k+\dots +\lambda _n$. The difficulty here is that there is a so-called *eigenvalue repulsion* phenomenon for Hermitian random matrices: the eigenvalues “want to be separated from each other”, which has the effect of “locking the spectrum in place”. Specifically (at least in the case where *m* and *n* are approximately equal), for any bounded test function *f*, the fluctuations of $f(\lambda _1)+\dots +f(\lambda _n)$ are tiny, of comparable size to the eigenvalues themselves (and so there is no chance of describing the fluctuations in $\lambda _k+\dots +\lambda _n$ via a central limit theorem for linear statistics of the form $f(\lambda _1)+\dots +f(\lambda _n)$).

Instead, we take advantage of the relationship between majorisation and convex functions: the *Hardy–Littlewood–Pólya theorem* says that if $(\lambda _k^{(1)})_{k=1}^n$ majorises $(\lambda _k^{(2)})_{k=1}^n$, then for any convex function $f:\mathbb R \rightarrow \mathbb R$ we have

$$\begin{aligned} f(\lambda _1^{(1)})+\dots +f(\lambda _n^{(1)})\ge f(\lambda _1^{(2)})+\dots +f(\lambda _n^{(2)}). \end{aligned}$$

So, we choose a sequence of convex test functions $f_1,\dots ,f_\ell$ which “emphasise distinct parts of the spectrum” and whose corresponding statistics therefore fluctuate almost independently. Specifically, for each *i*, we choose the function $f_i$ to grow much more rapidly than $f_1,\dots ,f_{i-1}$, and therefore focus much more strongly on the upper edge (the so-called *soft edge*) of the spectrum. For simplicity, we will ultimately take the functions $f_j$ to be polynomials of rapidly growing degree. We can then apply a general multivariate central limit theorem (with an adjustment for trace-normalisation) to the ℓ different statistics of the form $f_i(\lambda _1)+\dots +f_i(\lambda _n)$, and via analysis of certain integrals we can upper-bound the covariances between these statistics. The upshot is that we are able to show that the majorisation probability is at most about $2^{-\ell }$; since we can take ℓ arbitrarily large, the desired result follows.

The proof of Theorem 1.3 is completely different. Since the trace is extremely well-concentrated, it suffices to prove a version of Theorem 1.3 for eigenvalues of the *non-trace-normalised* complex Wishart–Laguerre ensemble. Writing $\mu _1,\dots ,\mu _n$ for these non-normalised eigenvalues, our approach is to prove that each individual eigenvalue $\mu _k$ is tightly concentrated (in a multiplicative sense) around its expected value $\mathbb E\mu _k$. Our proof of this fact relies on two ingredients: first, by Weyl’s inequalities, $\mu _k^{1/2}$ can be interpreted as a 1-Lipschitz function (with respect to the Euclidean norm) of a Gaussian random vector, so it follows from the Gaussian concentration inequality of Sudakov–Tsirelson and Borell that $\mu _k$ is well-concentrated in an *additive* sense. Second, since $m \ge n + C\sqrt{n\log n}$, it follows from known estimates on the smallest singular value of random matrices that $\mathbb {E}[\mu _{n}^{1/2}]$ is sufficiently large to convert the additive error to multiplicative error. The second ingredient is the only place where we use the separation between *m* and *n*; as we discuss in Remark 1.4, this is necessary.

## Proof of Nielsen’s Conjecture

In this section we present the proof of Theorem 1.1 (Nielsen’s conjecture), modulo some analytic estimates that will be deferred to Sect. 5.

Theorem 1.1 concerns parameters *m*, *n* which both tend to infinity, without making any assumption about the relationship between *m* and *n*. However, it is straightforward to reduce to the case where m≥n (by the symmetry between *m* and *n*), and it is then straightforward to reduce to the case where m/n→c for some constant $c\in [1,\infty ]$ (by compactness). There are certain complications in the c=∞ (“imbalanced”) case that are not present in the c<∞ (“approximately balanced”) case, so we choose to present these two cases separately.

### Majorisation and convexity

First, as discussed in section 1.3, it is crucial that instead of working with majorisation directly, we can work with convex test functions. To this end, we need the *Hardy–Littlewood–Pólya* theorem (appearing, for example, as [32, p. 259, Theorem B]).

#### Theorem 2.1

Consider any real numbers $\lambda _1^{(1)}\ge \dots \ge \lambda _n^{(1)}$ and $\lambda _1^{(2)}\ge \dots \ge \lambda _n^{(2)}$, such that

$$\begin{aligned} \lambda _k^{(1)}+ \dots + \lambda _n^{(1)}\le \lambda _k^{(2)}+ \dots + \lambda _n^{(2)}\text { for all }k\in \{1,\dots ,n-1\} \end{aligned}$$

$$\begin{aligned} \quad \text {and}\quad \lambda _1^{(1)}+ \dots + \lambda _n^{(1)}=\lambda _1^{(2)}+ \dots + \lambda _n^{(2)}. \end{aligned}$$

Then for any convex function $f:\mathbb R\rightarrow \mathbb R$, we have

$$\begin{aligned} f(\lambda _1^{(1)})+\dots +f(\lambda _n^{(1)})\le f(\lambda _1^{(2)})+\dots +f(\lambda _n^{(2)}). \end{aligned}$$

In particular, to show that a sequence $(\lambda _k^{(1)})_{k=1}^{n}$ is *not* majorised by $(\lambda _k^{(2)})_{k=1}^{n}$, it suffices to exhibit a convex function *f* for which $\sum _{k=1}^{n} f(\lambda _k^{(1)}) > \sum _{k=1}^{n} f(\lambda _k^{(2)})$. To prove Theorem 1.1, we will consider an explicit sequence of convex test functions $f_i$, and show that the events of the form $\sum _{k=1}^{n} f_i(\lambda _k^{(1)}) > \sum _{k=1}^{n} f_i(\lambda _k^{(2)})$ (for different *i*) are nearly independent from each other, meaning that it is very likely that at least one of these events holds (and therefore $(\lambda _k^{(1)})_{k=1}^{n}$ is likely not majorised by $(\lambda _k^{(2)})_{k=1}^{n}$).

### A central limit theorem for linear statistics, in the approximately balanced case

The other key general ingredient we will need is a (joint) central limit theorem for linear statistics of the complex Wishart–Laguerre spectrum. To start with, we present a central limit theorem that is suitable for the c<∞ case (i.e., the case where *m* and *n* are approximately balanced). The statement of our central limit theorem requires some preparation.

#### Definition 2.2

For continuous functions $f,g:\mathbb R\rightarrow \mathbb R$, define

$$\begin{aligned}\Gamma (f,g)&=\frac{1}{\pi ^2} \int _{-1}^{1}\int _{-1}^{1} \left( \frac{f(x) - f(y)}{x-y} \right) \left( \frac{g(x) - g(y)}{x-y} \right) \frac{1 - xy}{\sqrt{1 - x^2} \sqrt{1 - y^2}}\,dx\,dy.\end{aligned}$$

Then, for $c \in [1,\infty )$, let $a_{\pm } = (1 \pm \sqrt{c})^2$ and $\Phi _c(x) = 2\sqrt{c} x + c + 1$, and for continuous $f,g:\mathbb R\rightarrow \mathbb R$, define

$$\gamma _c(f) = \frac{1}{2\pi } \int _{a_-}^{a_+} f(x)\frac{\sqrt{(a_+ - x)(x - a_-)}}{x} \,dx,\qquad \Gamma _c(f,g) = \Gamma (f\circ \Phi _c,g\circ \Phi _c).$$

Also, let F be the set of all continuously differentiable functions $f:\mathbb R\rightarrow \mathbb R$ with “sub-exponential growth”, in the sense that $(\log |f(x)|)/|x|\rightarrow 0$ when $|x|\rightarrow \infty$.

#### Theorem 2.3

Fix functions $f_1,\dots ,f_\ell \in \mathcal F$ and let $\mu _1,\dots ,\mu _n$ be the eigenvalues of a random matrix sampled from the complex Wishart–Laguerre ensemble with parameters *m*, *n*. Suppose $m,n \rightarrow \infty$ in such a way that $m/n \rightarrow c \in [1,\infty )$. For each $i\in \{1,\dots ,\ell \}$ let

$$\begin{aligned} X_i= \sum _{k = 1}^n f_i(\mu _k/n). \end{aligned}$$

Then the following two asymptotic properties hold.

1. For each $i\in \{1,\dots ,\ell \}$, we have the $L^1$-convergence
  $$\begin{aligned} \mathbb {E}\big |X_i/n- \gamma _c(f_i)\big |\rightarrow 0. \end{aligned}$$
2. We have the multivariate convergence in distribution
  $$\begin{aligned} \big (X_1-\mathbb EX_1,\;\dots ,\;X_\ell -\mathbb EX_\ell )\overset{d}{\rightarrow }\mathcal N(\textbf{0},\Sigma ), \end{aligned}$$
  where the limiting covariance matrix $\Sigma \in \mathbb R^{\ell \times \ell }$ has (*i*, *j*)-entry equal to $\Gamma _c(f_i,f_j)$.

Theorem 2.3 can be proved by combining a few different results in the literature. The asymptotic formulas for the means in (1) go back to the seminal work of Marchenko and Pastur [24], and a central limit theorem was first proved by Arharov [2]. However, Arharov did not provide formulas for the covariances; for the covariance formulas in (2), we use a result of Lytova and Pastur [23]. There are three differences between these results and Theorem 2.3: first, our result is valid for a larger class of test functions; second, in (1), we obtain convergence in mean (whereas previous results obtain almost-sure convergence or convergence in probability—the distinction will be important for our application), and third, our result is a multivariate central limit theorem (whereas most previous results are univariate). In Sect. Appendix A we show how to deduce Theorem 2.3 from previous results (the deduction is standard, but we include this for completeness).

#### Remark

It is perhaps more common to study the *real* Wishart–Laguerre ensemble, which is the distribution of $A A^\intercal$ when $A\in \mathbb R^{n\times m}$ is a random matrix with independent *real* standard Gaussian entries. The distinction between the real and complex case does not affect Theorem 2.3(1), and affects the limiting covariances in Theorem 2.3 (2) by a factor of exactly 2 (i.e., the covariances are twice as large in the real case as in the complex case); see e.g. [23, Remark 4.1].

In our proof of Theorem 1.1, the only reason we need Theorem 2.3 is for the following corollary, providing a central limit theorem for sums of powers of trace-normalised eigenvalues.

#### Corollary 2.4

Let $\mu _1,\dots ,\mu _n$ be the eigenvalues of a random matrix sampled from the complex Wishart–Laguerre ensemble with parameters m≥n and let $\lambda _1,\ldots ,\lambda _n$ denote the trace-normalised eigenvalues. Fix ℓ, and for each $i\in \{1,\dots ,\ell \}$ let

$$\begin{aligned} X_i= \sum _{k = 1}^n (\mu _k/n)^i \quad \text { and } \quad Y_i = \sum _{k = 1}^n \lambda _k^i. \end{aligned}$$

Suppose $n,m\rightarrow \infty$ in such a way that m/n→c, for some fixed $c\in [1,\infty )$. Then we have multivariate convergence in distribution

$$\begin{aligned} \Bigg ( n\bigg (Y_i \cdot \frac{(\mathbb {E}X_1)^i}{\mathbb {E}X_i} - 1\bigg ) \Bigg )_{i \le \ell } \overset{d}{\rightarrow }\left( \frac{Z_i}{\gamma _c(x^i)} - i \frac{Z_1}{\gamma _c(x)}\right) _{i \le \ell } \end{aligned}$$

where $(Z_i)_{i \le \ell } \sim \mathcal {N}(\textbf{0}, \Sigma )$ for a matrix $\Sigma \in \mathbb R^{\ell \times \ell }$ with (*i*, *j*)-entry $\Gamma _c(x^i,x^j)$.

(Here we are abusing notation slightly, writing $x^i$ to indicate the function $x\mapsto x^i$).

#### Proof

Writing $\widetilde{Z}_i = X_i - \mathbb {E}X_i$, note that by Theorem 2.3 we have convergence in distribution $(\widetilde{Z}_i)_{i \le \ell } \rightarrow (Z_i)_{i \le \ell }.$ We may then expand

2.1 $$\begin{aligned} Y_i \cdot \frac{(\mathbb {E}X_1)^i}{\mathbb {E}X_i} = \frac{X_i}{X_1^i} \cdot \frac{(\mathbb {E}X_1)^i}{\mathbb {E}X_i} = \left( 1 + \frac{\widetilde{Z}_i}{\mathbb {E}X_i} \right) \left( 1 + \frac{\widetilde{Z}_1}{\mathbb {E}X_1}\right) ^{-i} = 1 + \frac{\widetilde{Z}_i}{\mathbb {E}X_i} - \frac{i \widetilde{Z}_1}{\mathbb {E}X_1} + O(n^{-2}). \end{aligned}$$

Here, asymptotics are “in probability”, treating *c*, *i* as constants: the notation $O(n^{-2})$ denotes a random variable $E_{c,i}$ such that $n^2 E_{c,i}$ is bounded in probability (as $n\rightarrow \infty$, holding *c*, *i* fixed). This estimate for $E_{c,i}$ follows from the fact that the random variables $\widetilde{Z}_i$ are bounded in probability (which is a consequence of Theorem 2.3 (2)) and the fact that $\mathbb {E}X_i = n\gamma _{c}(x^i) + o(n)$ has order of magnitude *n* (here we used Theorem 2.3(1), and the observation that $\gamma _c(x^i) \in (0,\infty )$ for all $c \in [1,\infty )$ and i≥1).

Rearranging (2.1) and using again that $\mathbb {E}X_i = n\gamma _c(x^i) + o(n)$ completes the proof.

□

#### Remark

Our choice of test functions $x\mapsto x^i$ is mainly to keep the proof of Corollary 2.4 (and its counterpart for the imbalanced case, Corollary 2.8) as simple as possible. If one were interested in optimising the quantitative aspects of our proof strategy, one should presumably consider alternative test functions.

### The covariance structure, and putting the pieces together

In order to use Corollary 2.4, we will need some control over the quantities $\gamma _c$ and $\Gamma _c$, which describe the limiting means and covariances of our test functions. This is the content of the next lemma, whose proof we defer to Sect. 5. Recall the definition of $a_+$ from Definition 2.2.

#### Lemma 2.5

Fix $c \in [1,\infty )$. For $i \in \mathbb {N}$, let $h_i(x) = (x/a_+)^i$.

1. We have $$\lim _{i \rightarrow \infty } i\gamma _c( h_i ) = 0$$.
2. The limit $\alpha _c=\lim _{i \rightarrow \infty } \Gamma _c(h_i,h_i)$ exists, and satisfies $\alpha _c>0$.
3. We have $$\lim _{A \rightarrow \infty } \lim _{i \rightarrow \infty } \Gamma _c(h_i, h_{Ai}) = 0$$.

Recall the random variables $Y_i=\sum _{k=1}^n \lambda _k^i$ from Corollary 2.4, which can be interpreted as linear statistics associated with convex test functions $f_i:x\mapsto x^i$. The estimates in Lemma 2.5 can be interpreted as saying that the fluctuations of $Y_i$ and $Y_j$ are nearly independent, as long as *i* is very large and *j* is much larger than *i* (specifically, (1) can be used to show that correlations due to trace-normalisation are not very impactful, and (2) and (3) can then be used together to show that the covariance between the non-trace-normalised random variables $X_i$ and $X_j$ is negligible compared to the variances of $X_i$ and $X_j$ individually).

#### Remark

One can compute the relevant integrals in Lemma 2.5, and apply them to prove Theorem 1.1, without any intuitive understanding of *why* estimates of this type should hold. However, roughly speaking, the picture to keep in mind is that if *j* is much larger than *i*, then the largest eigenvalues (and their fluctuations) contribute much more strongly to $Y_j$ than to $Y_i$. That is to say, $Y_j$ is dominated by a few very large eigenvalues, which play only a negligible role in $Y_i$. So, intuitively speaking, Lemma 2.5 corresponds to the fact that different parts of the Wishart–Laguerre spectrum have nearly independent fluctuations, and the sizes of these fluctuations do not significantly decay as we approach the upper edge of the spectrum (the latter is important as there is a “competition” between these fluctuations and the fluctuations due to trace-normalisation). We remark that the *lower* edge of the spectrum does not enjoy these properties: since all eigenvalues are always at least zero, the fluctuations of the smallest eigenvalues are more constrained. The upper and lower edges of the spectrum are sometimes called the “soft edge” and “hard edge” for this reason.

Given the preparations in this section so far, we can now prove Nielsen’s conjecture in the case where m/n→c for $c\in [1,\infty )$.

#### Proof of Theorem 1.1

, in the case $m/n\rightarrow c\in [1,\infty )$

Let $\mathcal {E}_{\textrm{maj}}$ be the event that $\lambda _k^{(1)}+\dots +\lambda _n^{(1)}\le \lambda _k^{(2)}+\dots +\lambda _n^{(2)}$ for all k≤n. Recall that we wish to show that $\mathbb {P}[\mathcal {E}_{\textrm{maj}}] \rightarrow 0$, when $m,n \rightarrow \infty$ in such a way that $m/n\rightarrow c\in [1,\infty )$.

For $s \in \{1,2\}$ define $Y_i^{(s)} = \sum _{j = 1}^n (\lambda _j^{(s)})^i.$ By convexity of the functions $x \mapsto x^i$ on [0,∞), and Theorem 2.1, we see that on the event $\mathcal {E}_{\textrm{maj}}$ we must have $Y_i^{(1)} \le Y_i^{(2)}$. By Corollary 2.4 this implies that for any ℓ, we have

$$\begin{aligned} \limsup _{n \rightarrow \infty } \mathbb {P}[\mathcal {E}_{\textrm{maj}}] \le \mathbb {P}\left[ Z_i^{(1)} \le Z_i^{(2)} - \frac{i \gamma _c(x^i)}{\gamma _c(x)}\big (Z_1^{(2)} - Z_1^{(1)} \big ) \, \text{ for } \text{ all } i \le \ell \right] , \end{aligned}$$

where $(Z_i^{(1)})_{i \le \ell }$ and $(Z_i^{(2)})_{i \le \ell }$ are independent Gaussian vectors as defined in Corollary 2.4.

Since the left-hand side is independent of ℓ, we may take $\ell \rightarrow \infty$. Writing

$$ \mathcal E_\ell =\left\{ Z_i^{(1)} \le Z_i^{(2)} - \frac{i \gamma _c(x^i)}{\gamma _c(x)}\bigl (Z_1^{(2)} - Z_1^{(1)} \bigr )\, \text {for all } i \le \ell \right\} , $$

note that the events $\mathcal E_\ell$ are decreasing in ℓ. Therefore, by continuity from above,

$$\begin{aligned} \limsup _{n \rightarrow \infty } \mathbb {P}[\mathcal {E}_{\textrm{maj}}] \le \lim _{\ell \rightarrow \infty }\mathbb {P}[\mathcal E_\ell ] = \mathbb {P}\left[ Z_i^{(1)} \le Z_i^{(2)} - \frac{i \gamma _c(x^i)}{\gamma _c(x)}\bigl (Z_1^{(2)} - Z_1^{(1)} \bigr )\, \text {for all } i\right] . \end{aligned}$$

Define $W_i^{(s)} = Z_i^{(s)} / a_+^i$, and let $h_i(x)=(x/a_+)^i$ as in Lemma 2.5. Note that the covariance between $W_i^{(s)}$ and $W_j^{(s)}$ is precisely $\Gamma _c(h_i,h_j)$, and note that

$$\begin{aligned}&\mathbb {P}\left[ Z_i^{(1)} \le Z_i^{(2)} - \frac{i \gamma _c(x^i)}{\gamma _c(x)}\bigl (Z_1^{(2)} - Z_1^{(1)} \bigr )\, \text {for all } i\right] \\&= \mathbb {P}\left[ W_i^{(1)} \le W_i^{(2)} - \frac{i \gamma _c(h_i)}{\gamma _c(x)}\bigl (Z_1^{(2)} - Z_1^{(1)}\bigr )\, \text {for all } i \right] . \end{aligned}$$

Now, for each $j,q,A \in \mathbb {N}$, set $i_j(q,A) = q A^j$. So, by Lemma 2.5, as we send $A,q\rightarrow \infty$ (where $A\rightarrow \infty$ much more rapidly than $q\rightarrow \infty$), we observe:

1. $$i_j(q,A) \gamma _c(h_{i_j(q,A)})\rightarrow 0$$;
2. the variances of the individual $W_{i_j(q,A)}^{(s)}$ tend to $\alpha _c>0$;
3. the covariances between different $W_{i_j(q,A)}^{(s)}$ tend to zero.

Fix $N\in \mathbb N$. For each *q*, *A*, the random vector

$$ \Bigl (W_{i_j(q,A)}^{(1)},W_{i_j(q,A)}^{(2)}\Bigr )_{j\le N} $$

is centred Gaussian. By the three observations above, its covariance matrix converges entrywise, as first $q\rightarrow \infty$ and then $A\rightarrow \infty$, to the diagonal matrix $\alpha _c I_{2N}$. Since a centred Gaussian law is determined by its covariance matrix, it follows that

$$ \Bigl (W_{i_j(q,A)}^{(1)},W_{i_j(q,A)}^{(2)}\Bigr )_{j\le N} \overset{d}{\rightarrow }\Bigl (\zeta _j^{(1)},\zeta _j^{(2)}\Bigr )_{j\le N}, $$

where $(\zeta _j^{(1)},\zeta _j^{(2)})_{j\le N}$ are i.i.d. real Gaussians with mean zero and variance $\alpha _c$.

Also, if we define

$$ b_{j,q,A}=\frac{i_j(q,A)\gamma _c(h_{i_j(q,A)})}{\gamma _c(x)}, $$

then by (1) we have $\max _{j\le N}|b_{j,q,A}|\rightarrow 0$ as first $q\rightarrow \infty$ and then $A\rightarrow \infty$. Since $Z_1^{(2)} - Z_1^{(1)}$ is bounded in probability, it follows that

$$ \Bigl (b_{j,q,A}\bigl (Z_1^{(2)} - Z_1^{(1)}\bigr )\Bigr )_{j\le N}\rightarrow \textbf{0} \qquad \text {in probability.} $$

Therefore, by Slutsky’s theorem,

$$ \Bigl (W_{i_j(q,A)}^{(2)} - W_{i_j(q,A)}^{(1)} - b_{j,q,A}\bigl (Z_1^{(2)} - Z_1^{(1)}\bigr )\Bigr )_{j\le N} \overset{d}{\rightarrow }\Bigl (\zeta _j^{(2)} - \zeta _j^{(1)}\Bigr )_{j\le N}. $$

Since the limiting law is absolutely continuous, the boundary of the orthant event has probability zero, and hence

$$\begin{aligned} \mathbb {P}&\left[ W_i^{(1)} \le W_i^{(2)} - \frac{i \gamma _c(h_i)}{\gamma _c(x)}\bigl (Z_1^{(2)} - Z_1^{(1)}\bigr )\, \text {for all } i \right] \\&\le \lim _{A \rightarrow \infty }\lim _{q \rightarrow \infty } \mathbb {P}\left[ W_{i_j(q,A)}^{(1)} \le W_{i_j(q,A)}^{(2)} - \frac{i_j(q,A) \gamma _c(h_{i_j(q,A)})}{\gamma _c(x)}\bigl (Z_1^{(2)} - Z_1^{(1)}\bigr )\, \text {for all } j \le N \right] \\&= \mathbb {P}\big [\zeta _j^{(1)} \le \zeta _j^{(2)}\, \text {for all } j \le N\big ] = 2^{-N}. \end{aligned}$$

Taking $N\rightarrow \infty$ completes the proof. □

### The imbalanced case

In this subsection, we present analogues of Theorem 2.3, Corollary 2.4, and Lemma 2.5 for the “imbalanced” case where $m/n\rightarrow \infty$. We then deduce the statement of Theorem 1.1 in this case, and provide the details for how to deduce the full statement of Theorem 1.1 from the approximately-balanced and imbalanced cases.

The main technical difficulty in the imbalanced case is that the spectrum of the complex Wishart–Laguerre ensemble is concentrated around *m* (i.e., typically all eigenvalues are of the form m-o(m)). This means that linear statistics $Y_i=\sum _{k=1}^{n} \lambda _k^i$ of the type considered in Corollary 2.4 have variance *o*(1), and their limiting distribution is degenerate.

The root of the problem is that the normalisation in Theorem 2.3 (considering statistics of the form $\sum _{k=1}^nf(\mu _k/n)$) is not appropriate in the imbalanced regime, and to get a sensible central limit theorem, one should renormalise with an “additive shift”: namely, one should consider statistics of the form $\sum _{k=1}^nf\big ((\mu _k-m)/\sqrt{nm}\big )$. The following central limit theorem is an analogue of Theorem 2.3 with this renormalisation.

#### Definition 2.6

Recall the set of functions F with “sub-exponential growth”, and the notation Γ(f,g), from Definition 2.2. For a continuous function $f:\mathbb R\rightarrow \mathbb R$, let

$$\begin{aligned} \gamma (f) = \frac{1}{\pi }\int _{-1}^1 f(x) \sqrt{1 - x^2}\,dx. \end{aligned}$$

#### Theorem 2.7

Fix functions $f_1,\dots ,f_\ell \in \mathcal F$ and let $\mu _1,\dots ,\mu _n$ be the eigenvalues of a random matrix sampled from the complex Wishart–Laguerre ensemble with parameters *m*, *n*. Suppose $m,n \rightarrow \infty$ in such a way that $m/n \rightarrow \infty$. For each $i\in \{1,\dots ,\ell \}$ let

$$\begin{aligned} X_i= \sum _{k = 1}^n f_i\left( \frac{\mu _k - m}{2\sqrt{nm}}\right) . \end{aligned}$$

Then the following two asymptotic properties hold.

1. For each $i\in \{1,\dots ,\ell \}$, we have the $L^1$-convergence
  $$\begin{aligned} \mathbb {E}\big |X_i/n- \gamma (f_i)\big |\rightarrow 0. \end{aligned}$$
2. We have the multivariate convergence in distribution
  $$\begin{aligned} \big (X_1-\mathbb EX_1,\;\dots ,\;X_\ell -\mathbb EX_\ell )\overset{d}{\rightarrow }\mathcal N(\textbf{0},\Sigma ), \end{aligned}$$
  where $\Sigma \in \mathbb R^{\ell \times \ell }$ is a matrix with (*i*, *j*)-entry $\Gamma (f_i,f_j)$.

As for Theorem 2.3, we can prove Theorem 2.7 by combining various results in the literature (the deduction can again be found in Sect. Appendix A). The two normalisations used in Theorem 2.3, 2.7 correspond to the two standard asymptotic spectral regimes for Wishart matrices. When $m/n\rightarrow c<\infty$, the empirical spectral measure of $(\mu _k/n)_{k=1}^n$ converges to the Marchenko–Pastur law with parameter *c*, which underlies Theorem 2.3. When $m/n\rightarrow \infty$, after centering at *m* and scaling by $2\sqrt{mn}$, the empirical spectral measure converges to the semicircular law, which underlies Theorem 2.7. In this sense, Theorem 2.7 is the $c\rightarrow \infty$ counterpart of Theorem 2.3. Specifically, the formulas for the means in Theorem 2.7(1) are essentially due to Bai and Yin [5], and a central limit theorem in this regime was proved independently by Bao [6] and by Chen and Pan [9]. See also Nechita [27] for related results on random density matrices.

#### Remark

We have stated Theorem 2.7 only for the case $m/n\rightarrow \infty$ (as we have already handled the case where *m*/*n* converges to a finite limit), but we remark that it would be possible to state a more general (albeit somewhat complicated) central limit theorem that holds for all $m,n\rightarrow \infty$, and use this to give a unified proof of Theorem 1.1 without a case distinction.

Now, as an analogue of Corollary 2.4, we next deduce a corollary of Theorem 2.7 for powers of (suitably shifted) trace-normalised eigenvalues. Due to the shifting, the deduction is somewhat more complicated than for Corollary 2.4.

#### Corollary 2.8

Let $\mu _1,\dots ,\mu _n$ be the eigenvalues of a random matrix sampled from the complex Wishart–Laguerre ensemble with parameters *n*, *m* and let $\lambda _1,\ldots ,\lambda _n$ denote the trace-normalised eigenvalues. Fix ℓ, and for each $i\in \{1,\dots ,2\ell \}$ let

$$\begin{aligned} X_i= \sum _{k = 1}^n \left( \frac{\mu _k -m}{2\sqrt{mn}}\right) ^i \quad \text { and } \quad Y_i = \sum _{k = 1}^n \left( \lambda _k - \frac{1}{n}\right) ^i. \end{aligned}$$

Suppose $n,m\rightarrow \infty$ in such a way that $m/n\rightarrow \infty$. Then we have multivariate convergence in distribution

$$\begin{aligned} \Bigg ( n\bigg (Y_{2i} \cdot \frac{(mn/4)^{i}}{(\mathbb {E}X_{2i})} - 1\bigg ) \Bigg )_{i \le \ell } \overset{d}{\rightarrow }\left( \frac{Z_{2i}}{\gamma (x^{2i})}\right) _{i \le \ell } \end{aligned}$$

where $(Z_{2i})_{i \le \ell } \sim \mathcal {N}(\textbf{0}, \Sigma )$ for a matrix $\Sigma \in \mathbb R^{\ell \times \ell }$ with (*i*, *j*)-entry $\Gamma (x^{2i},x^{2j})$.

In our proof of Corollary 2.8, we will need the following basic fact.

#### Fact 2.9

In the setting of Corollary 2.8 we have $\mathbb {E}[\mu _1+\dots +\mu _n]=mn$.

#### Proof

Let $G\in \mathbb C^{n\times m}$ be a random matrix with independent standard complex Gaussian entries, in such a way that $\mu _1,\dots ,\mu _n$ are the eigenvalues of $GG^\dagger$. Then, we have

$$\begin{aligned} \mu _1+\dots +\mu _n=\sum _{i=1}^n\sum _{j=1}^m |G_{ij}|^2, \end{aligned}$$

and the desired result follows (recalling that the entries $G_{ij}$ are complex standard Gaussian). □

#### Proof of Corollary 2.8

Throughout this proof, all implicit constants in asymptotic notation are allowed to depend on ℓ (i.e., we think of ℓ as a constant).

Writing $\widetilde{Z}_i = X_i - \mathbb {E}X_i$, by Theorem 2.7(2) we have the convergence in distribution $(\widetilde{Z}_i)_{i \le 2\ell } \rightarrow (Z_i)_{i \le 2\ell }.$ Then, writing $T=\mu _1+\dots +\mu _n$, note that

$$\begin{aligned} T = mn + 2 \sqrt{nm}\widetilde{Z}_1, \end{aligned}$$

using Fact 2.9. We may then write

2.2 $$\begin{aligned} Y_{2i} = T^{-2i} \sum _{k=1}^n \left( \mu _k - \frac{T}{n} \right) ^{2i} = \left( \frac{4}{mn}\right) ^i\left( 1 + \frac{2 \widetilde{Z}_1}{\sqrt{mn}} \right) ^{-2i} \sum _{k=1}^n \left( \frac{\mu _k - m}{2\sqrt{mn}} - \frac{\widetilde{Z}_1}{n}\right) ^{2i}. \end{aligned}$$

Since $\tilde{Z_1}$ is bounded in probability by Theorem 2.7(2), we have that

2.3 $$\begin{aligned} \left( 1 + \frac{2 \widetilde{Z}_1}{\sqrt{mn}} \right) ^{-2i} = 1 + O\left( \frac{1}{\sqrt{mn}}\right) = 1 + o\left( \frac{1}{n}\right) , \end{aligned}$$

where asymptotics are in probability.

Moreover, writing $\varepsilon = -\widetilde{Z}_1/n$, we expand

$$\begin{aligned} \sum _{k=1}^n \left( \frac{\mu _k - m}{2\sqrt{mn}} + \varepsilon \right) ^{2i}&= \sum _{k=1}^n \sum _{j = 0}^{2i} \left( {\begin{array}{c}2i\\ j\end{array}}\right) \left( \frac{\mu _k - m}{2\sqrt{mn}} \right) ^{2i - j}\varepsilon ^j \\&= \sum _{j = 0}^{2i}\left( {\begin{array}{c}2i\\ j\end{array}}\right) X_{2i - j} \varepsilon ^j = \sum _{j = 0}^{2i}\left( {\begin{array}{c}2i\\ j\end{array}}\right) \left( \mathbb {E}X_{2i - j} + \widetilde{Z}_{2i - j}\right) \varepsilon ^j\\&= \mathbb {E}X_{2i} + \widetilde{Z}_{2i} + \sum _{j = 1}^{2i}\left( {\begin{array}{c}2i\\ j\end{array}}\right) \widetilde{Z}_{2i-j}\varepsilon ^j + \sum _{j = 1}^{2i}\left( {\begin{array}{c}2i\\ j\end{array}}\right) (\mathbb {E}X_{2i-j})\varepsilon ^j. \end{aligned}$$

Now, recall from Theorem 2.7(1) that for each $r\le 2\ell$ we have

$$ \mathbb {E}X_r = n\gamma (x^r) + o(n). $$

In particular, for each $j\le \ell$ we have $\mathbb {E}X_{2j} = O(n)$, while $\mathbb {E}X_{2j-1} = o(n)$ since $\gamma (x^{2j-1})=0$. Also, by Theorem 2.7(2), each $\widetilde{Z}_r$ is bounded in probability, and hence ε=O(1/n) in probability.

Therefore every term in the two sums above is *o*(1) in probability. Indeed, if j≥1, then

$$ \widetilde{Z}_{2i-j}\varepsilon ^j = O(1)\cdot O(1/n^j)=o(1) $$

in probability. Also, if j=1 and 2i-j is odd, then

$$ (\mathbb {E}X_{2i-1})\varepsilon = o(n)\cdot O(1/n)=o(1) $$

in probability, while if j≥2, then

$$ (\mathbb {E}X_{2i-j})\varepsilon ^j = O(n)\cdot O(1/n^j)=o(1) $$

in probability. Since there are only finitely many such terms (with ℓ fixed), it follows that

2.4 $$\begin{aligned} \sum _{k=1}^n \left( \frac{\mu _k - m}{2\sqrt{mn}} + \varepsilon \right) ^{2i} = \mathbb {E}X_{2i} + \widetilde{Z}_{2i} + o(1), \end{aligned}$$

where the *o*(1) term is in probability. The desired result then follows from Equations (2.2) to (2.4). □

Finally, as an analogue of Lemma 2.5, we need the following technical estimates on Γ, which are proved in Sect. 5.

#### Lemma 2.10

1. The limit $\alpha =\lim _{i \rightarrow \infty } \Gamma (x^i,x^i)$ exists, and satisfies α>0.
2. We have $$\lim _{A \rightarrow \infty } \lim _{i \rightarrow \infty } \Gamma (x^i, x^{Ai}) = 0. $$

We can now complete the proof of Theorem 1.1, supplementing the approximately balanced case in the last subsection with similar considerations in the imbalanced case (using Corollary 2.8, 2.10).

#### Proof of Theorem 1.1

As before, let $\mathcal {E}_{\textrm{maj}}$ be the event that $\lambda _k^{(1)}+\dots +\lambda _n^{(1)}\le \lambda _k^{(2)}+\dots +\lambda _n^{(2)}$ for all k≤n. We wish to show that $\mathbb {P}[\mathcal {E}_{\textrm{maj}}] \rightarrow 0$ as $m,n \rightarrow \infty$.

First, note that we can restrict our attention to the case where m≥n. Indeed, recall that the complex Wishart–Laguerre ensemble with parameters *n*, *m* is the distribution of a random matrix of the form $A A^\dagger$, where $A\in \mathbb C^{n\times m}$ is a matrix with standard complex Gaussian entries. Switching the roles of *m* and *n* is equivalent to considering the matrix $A^\dagger A$, which has the same nonzero eigenvalues as $AA^\dagger$ (if n≤m, this switch merely introduces m-n additional zero eigenvalues) and this is the only relevant information for the event $\mathcal {E}_{\textrm{maj}}$.

Second, note that if Theorem 1.1 were not true, then there would be some sequence of parameter-pairs $(n,m_n)$, with $m_n \ge n\rightarrow \infty$, such that $\limsup _{n\rightarrow \infty }\mathbb P[\mathcal {E}_\mathrm{{maj}}]>0$. By compactness of [1,∞], there would then be an infinite subsequence of values of *n* along which $m_n/n\rightarrow c$ for some $c\in [1,\infty ]$. So, in proving Theorem 1.1 we may assume that $m_n/n\rightarrow c$ for some $c\in [1,\infty ]$. In Sect. 2.3 we have already handled the case where c<∞, we may (and do) assume that $m/n\rightarrow \infty$.

For $s \in \{1,2\}$, let $Y^{(s)}_{i} = \sum _{k=1}^{n}(\lambda _k^{(s)} - 1/n)^{i}$. By convexity of the functions $x \mapsto (x-1/n)^{2i}$, it follows from Corollary 2.8, 2.1 (as in the approximately balanced case) that

$$\begin{aligned} \limsup _{n\rightarrow \infty }\mathbb {P}[\mathcal {E}_{\textrm{maj}}] \le \mathbb {P}\big [Z_{2i}^{(1)} \le Z_{2i}^{(2)} \text { for all } i\big ], \end{aligned}$$

where $(Z_{i}^{(1)})_i$ and $(Z_{i}^{(2)})_i$ are independent vectors distributed as in Corollary 2.8.

Proceeding as in the approximately balanced case, but now using Lemma 2.10, it follows that if we let $(\zeta _j^{(1)}, \zeta _j^{(2)})$ denote i.i.d. (real) Gaussians with mean zero and variance α, then

$$\begin{aligned} \mathbb {P}[Z_i^{(1)} \le Z_{i}^{(2)}\text { for all }i] \le \lim _{N \rightarrow \infty } \mathbb {P}\big [\zeta _j^{(1)} \le \zeta _j^{(2)}\text { for all }j\le N\big ] = 0. \end{aligned}$$

□

## The Uniform Measure

In this section we prove Theorem 1.2, for the uniform measure on the simplex. The starting point is that if $(X_{1},\dots ,X_{n})$ is uniformly distributed on the simplex, then the decreasing rearrangement $(\lambda _{1},\dots ,\lambda _{n})$ has a *closed form description* in terms of independent random variables, as follows.

### Theorem 3.1

Let $Z_{1},\dots ,Z_{n}$ be i.i.d. exponential random variables with mean 1. Then the normalised vector

$$ \frac{1}{Z_{1}+\dots +Z_{n}}(Z_{1},\dots ,Z_{n}) $$

is uniformly distributed in the simplex $\Delta _{n-1}$.

### Theorem 3.2

Let $Z_{1},\dots ,Z_{n}$ be i.i.d. exponential random variables with mean 1, and let $Z_{(1)}<\dots <Z_{(n)}$ be their order statistics (i.e., $(Z_{(n-i+1)})_{i=1}^{n}$ is the decreasing rearrangement of $(Z_{i})_{i=1}^{n}$). Then $(Z_{(i)})_{i=1}^{n}$ has the same distribution as

$$ \left( \frac{Z_{1}}{n},\quad \frac{Z_{1}}{n}+\frac{Z_{2}}{n-1},\quad \frac{Z_{1}}{n}+\frac{Z_{2}}{n-1}+\frac{Z_{3}}{n-2},\quad \dots ,\quad \frac{Z_{1}}{n}+\dots +\frac{Z_{n}}{1}\right) . $$

Fact 3.2, 3.1 are both classical. In particular, Fact 3.1 is a direct consequence of [15, Chapter V, Theorems 2.1 and 2.2], and Fact 3.2 can be found in [15, Chapter V, Section 3.3].

Now, before getting into the details, we briefly describe the plan to prove Theorem 1.2. First, by a standard concentration inequality (e.g. Bernstein’s inequality, see for example [32, Theorem 2.8.1 and Example 2.7.8]), sums of exponential random variables do not fluctuate very much, as follows.

### Theorem 3.3

Let $Z_{1},\dots ,Z_{N}$ be i.i.d. exponential random variables with mean 1, and let $U=a_{1}Z_{1}+\dots +a_{N}Z_{N}$ for some coefficients $a_1,\dots ,a_N\in \mathbb R$. There is an absolute constant c>0 such that

$$ \mathbb P\big [|U-\mathbb EU|\ge t\big ]\le \exp \left( -c\min \left( \frac{t^{2}}{a_{1}^{2}+\dots +a_{N}^{2}},\;\frac{t}{\max _{i}|a_{i}|}\right) \right) $$

for every t≥0.

Given Fact 3.3 (applied with $a_{1}=\dots =a_{n}=1$), we can approximate quantities of the form $\lambda _{k}+\dots +\lambda _{n}$ (as in the statement of Theorem 1.2) with sums of the form

$$ \sum _{j=1}^{n-k+1}\sum _{i=1}^{j}\frac{Z_{i}}{n-(i-1)} $$

If n-k is small, then each of the denominators n-i+1 is very close to *n* (i.e., the denominators are all almost the same), meaning that it suffices to understand the *iterated partial sums* of the form $\sum _{j=1}^{q}\sum _{i=1}^{j}Z_{i}$. There is a fairly substantial literature on this topic; in particular, we take advantage of the following powerful theorem of Dembo, Ding and Gao [14, Theorem 1.1], on *persistence probabilities* for iterated partial sums.

### Theorem 3.4

Let $(W_{i})_{i\in \mathbb N}$ be a sequence of i.i.d. random variables with zero mean and $0<\mathbb E W_{i}^{2}<\infty$. For $q\in \mathbb N$, let $S_{q}=\sum _{j=1}^{q}\sum _{i=1}^{j}W_{i}$. Then for any fixed $t\in \mathbb R$ we have

$$ \mathbb P\big [S_{q}<t\text { for all }q\le N\big ]= \Theta _t(N^{-1/4}). $$

We now provide the full details of the proof of Theorem 1.2.

### Proof of Theorem 1.2

Let $(Z_j)_{j=1}^n$ and $(Z_j')_{j=1}^n$ be i.i.d. sequences of standard exponential random variables, and let $(Z_{(j)})_{j=1}^n$ and $(Z_{(j)}')_{j=1}^n$ be their order statistics. Let $\mathcal E_{\textrm{maj}}$ be the event that

$$\begin{aligned} \frac{Z_{(1)}+\dots +Z_{(q)}}{Z_{(1)}+\dots +Z_{(n)}}\le \frac{Z_{(1)}'+\dots +Z_{(q)}'}{Z_{(1)}'+\dots +Z_{(n)}'} \end{aligned}$$

for all q≤n. Recalling Fact 3.1, our goal is to prove that $\mathbb P[\mathcal E_{\textrm{maj}}]\le O((n/\log n)^{-1/16})$.

By Fact 3.3, for a sufficiently large constant *C* we have $|Z_1+\dots +Z_n-n|\le C\sqrt{n\log n}$ with probability at least $1 - n^{-100}$. So,

$$\begin{aligned} \mathbb P[\mathcal E_{\textrm{maj}}] \end{aligned}$$

$$\begin{aligned} \le \mathbb P\left[ Z_{(1)}+\dots +Z_{(q)}\le \Bigg (1+3C\sqrt{\frac{\log n}{n}}\,\Bigg ) (Z_{(1)}'+\dots +Z_{(q)}')\text { for all }q\le n\right] +n^{-100}. \end{aligned}$$

Recalling Fact 3.2, it suffices to prove that

3.1 $$\begin{aligned} \mathbb {P}\left[ \sum _{j = 1}^q \sum _{i = 1}^j \frac{Z_i}{n-(i-1)} \le \Bigg (1 + 3C\sqrt{\frac{\log n}{n}}\,\Bigg )\sum _{j = 1}^q \sum _{i = 1}^j \frac{Z_i'}{n-(i-1)} \text { for all }q\le n \right] \le O((n/\log n)^{-1/16}). \end{aligned}$$

Let $Q=\lfloor c(n/\log n)^{1/4}\rfloor$ for some small constant c>0. By Fact 3.3, with probability at least $1-n^{-100}$ we have

$$ \sum _{j=1}^{Q}\sum _{i=1}^{j}\frac{Z_{i}'}{n-(i-1)}=\sum _{i=1}^{Q}\bigg (\frac{Q-(i-1)}{n-(i-1)}\cdot Z_{i}'\bigg )\le \frac{2Q^{2}}{n}\le \frac{2c^{2}}{\sqrt{n\log n}}, $$

and similarly, with probability at least $1-n^{-100}$ we have

$$ \left| \sum _{j=1}^{k}\sum _{i=1}^{j}\frac{Z_{i}-Z_{i}'}{n-(i-1)}-\sum _{j=1}^{k}\sum _{i=1}^{j}\frac{Z_{i}-Z_{i}'}{n}\right| $$

$$=\left| \sum _{i=1}^{k}\frac{i-1}{n}\cdot \frac{k-(i-1)}{n-(i-1)}\cdot (Z_{i}-Z_{i}')\right| \le \frac{1}{2n} $$

for all k≤Q. Assuming that *c* is sufficiently small (in terms of *C*), it follows that the probability in (3.1) is at most

$$\begin{aligned}&\mathbb P\left[ \sum _{j=1}^{q}\sum _{i=1}^{j}\frac{Z_{i}}{n-(i-1)}\le \sum _{j=1}^{q}\sum _{i=1}^{j}\frac{Z_{i}'}{n-(i-1)}+3C\sqrt{\frac{\log n}{n}}\cdot \frac{2c^{2}}{\sqrt{n\log n}}\text { for all }q\le Q\right] +n^{-100}\\&\qquad \le \mathbb P\left[ \sum _{j=1}^{q}\sum _{i=1}^{j}\frac{Z_{i}}{n-(i-1)}\le \sum _{j=1}^{q}\sum _{i=1}^{j}\frac{Z_{i}'}{n-(i-1)}+\frac{1}{2n}\text { for all }q\le Q\right] +n^{-100}\\&\qquad \le \mathbb P\left[ \sum _{j=1}^{q}\sum _{i=1}^{j}\frac{Z_{i}-Z_i'}{n}\le \frac{1}{n}\text { for all }q\le Q\right] +2n^{-100}\\&\qquad =\mathbb P\left[ \sum _{j=1}^{q}\sum _{i=1}^{j}(Z_{i}-Z_{i}')\le 1\text { for all }q\le Q\right] +2n^{-100}. \end{aligned}$$

This probability is at most $O(Q^{-1/4})=O((n/\log n)^{-1/16}),$ by Theorem 3.4. □

## Approximate LOCC-Convertibility

In this section, we prove Theorem 1.3 in the following, more precise, form.

### Theorem 4.1

Let $M^{(1)},M^{(2)}\in \mathbb C^{n\times n}$ be independent samples from the trace-normalised complex Wishart–Laguerre ensemble with parameters *n*, *m*. Let $\lambda _1^{(1)}\ge \dots \ge \lambda _n^{(1)}$ and $\lambda _1^{(2)}\ge \dots \ge \lambda _n^{(2)}$ denote the respective eigenvalues, and let

$$\begin{aligned} \Pi =\min _{1\le k\le n} \frac{\lambda _k^{(1)}+\lambda _{k+1}^{(1)}+\dots +\lambda _n^{(1)}}{\lambda _k^{(2)}+\lambda _{k+1}^{(2)}+\dots +\lambda _n^{(2)}}. \end{aligned}$$

For every $\varepsilon , \delta > 0$, there exists a constant $C(\varepsilon ,\delta ) > 0$ such that if $m \ge n + C(\varepsilon ,\delta )\sqrt{n\log {n}}$ and $n \ge C(\varepsilon ,\delta )$, then $\mathbb {P}[\Pi < 1 - \varepsilon ] \le \delta$.

In our proof of Theorem 4.1 we need a few general tools. First, we need concentration of the trace of the complex Wishart–Laguerre ensemble.

### Lemma 4.2

There is an absolute constant *c* such that the following holds. Let *M* be a sample from the complex Wishart–Laguerre distribution with parameters *n*, *m*. Then

$$\begin{aligned} \mathbb P\big [|\!\operatorname {tr}(M)-mn|>t\big ]\le \exp \!\Big (-c\min \big (t^2/(mn),t\big )\Big ). \end{aligned}$$

### Proof

As in the proof of Fact 2.9, we can write

$$\begin{aligned} \operatorname {tr}(M)=\sum _{i=1}^n\sum _{j=1}^m |G_{ij}|^2, \end{aligned}$$

where the $G_{ij}$ are independent standard complex Gaussians. The terms $|G_{ij}|^2$ are sub-exponential (see [39, Lemma 2.7.6]), so the desired result follows from Bernstein’s inequality (see [39, Theorem 2.8.1]). □

We also need the Gaussian concentration inequality for Lipschitz functions, due independently to Borell and Sudakov–Tsirelson (see [39, Definition 2.5.6 and Theorem 5.2.2]).

### Theorem 4.3

There is an absolute constant c>0 such that the following holds. Let $F:\mathbb R^n\rightarrow \mathbb R$ be a Lipschitz function (with respect to the Euclidean metric), with Lipschitz constant at most *r*. Let $X_1,\dots ,X_n\in \mathbb R$ be i.i.d. standard Gaussian random variables, and let $Y=F(X_1,\dots ,X_n)$. Then for any t≥0 we have

$$\begin{aligned} \mathbb P\big [|Y-\mathbb E Y|\ge t\big ]\le 2\exp (-ct^2/r^2). \end{aligned}$$

For a matrix $G \in \mathbb {C}^{n\times m}$, let $\sigma _k(G)$ denote the *k*-th largest singular value of *G* and let $\Vert G\Vert _\textrm{F} = \sqrt{\sum _{ij}|G_{ij}|^2}$ denote the Frobenius norm of *G*. The next ingredient we will need is a version of Weyl’s inequality. The following statement is a consequence of e.g. [38, Exercise 1.3.22(iv)], using the inequality $\Vert H\Vert _{\textrm{F}} = (\textrm{tr}(H^\dagger H))^{1/2} = (\sum _{i}\sigma _i(H)^2)^{1/2} \ge \sigma _1(H)$.

### Theorem 4.4

For any matrices $G,H\in \mathbb C^{n\times m}$, we have

$$\begin{aligned} |\sigma _k(G+H) - \sigma _k(G)| \le \Vert H\Vert _\textrm{F}. \end{aligned}$$

Finally, we need a lower bound on the expected least singular value of complex Gaussian rectangular matrices. The following estimate is a direct consequence of [22, Theorem 3.4], which is an adaptation of the main result of [33] to the complex case.

### Lemma 4.5

There is an absolute constant c>0 such that the following holds. Let *G* be an n×m matrix with independent complex standard Gaussian entries. Then, for all $1 \le k \le n$,

4.1 $$\begin{aligned} \mathbb {E}[\sigma _k(G)] \ge \mathbb {E}[\sigma _n(G)] \ge c\big (\sqrt{m} - \sqrt{n-1}\big ). \end{aligned}$$

Now we are ready to prove Theorem 4.1.

### Proof of Theorem 4.1

Throughout this proof, we treat $\varepsilon ,\delta$ as constants. In particular, implicit constants in asymptotic notation are allowed to depend on $\varepsilon ,\delta$. Our goal is to prove that $\mathbb {P}[\Pi < 1 - \varepsilon ] \le \delta$, as long as *n* is sufficiently large and $m\ge n+C\sqrt{n\log n}$ for large enough *C*.

Let $G^{(1)}, G^{(2)}$ be independent n×m matrices with independent complex standard Gaussian entries, and for $s\in \{1,2\}$ let $M^{(s)} = G^{(s)}(G^{(s)})^{\dagger } \in \mathbb {C}^{n\times n}$ denote the corresponding sample from the complex Wishart–Laguerre ensemble with parameters *n*, *m*. Let $\mu ^{(s)}_1 \ge \dots \ge \mu ^{(s)}_n \ge 0$ denote the eigenvalues of $M^{(s)}$; we may take $\lambda ^{(s)}_1 \ge \dots \ge \lambda ^{(s)}_n \ge 0$ to be the eigenvalues of $M^{(s)}/\operatorname {tr}(M^{(s)})$.

By Lemma 4.2, with probability at least 1-δ/2 we have $\operatorname {tr}(M^{(s)}) = mn + O(\sqrt{mn})$ for both $s \in \{1,2\}$. Therefore, it suffices to show that $\mathbb {P}[\Pi _{\mu } < 1 - \varepsilon ] \le \delta /2$, where

$$\begin{aligned} \Pi _{\mu } = \min _{1\le k\le n} \frac{\mu _k^{(1)}+\mu _{k+1}^{(1)}+\dots +\mu _n^{(1)}}{\mu _k^{(2)}+\mu _{k+1}^{(2)}+\dots +\mu _n^{(2)}}. \end{aligned}$$

We will, in fact, show the stronger statement that

$$\begin{aligned} \mathbb {P}\left[ \mu _k^{(1)} \in [(1-\varepsilon )\mu _k^{(2)}, (1+\varepsilon )\mu _k^{(2)}] \quad \text {for all }1 \le k \le n\right] \ge 1-\delta /2. \end{aligned}$$

By the union bound, it suffices to show that

4.2 $$\begin{aligned} \mathbb {P}\left[ \mu _k^{(1)} \in [(1-\varepsilon )\mu _k^{(2)}, (1+\varepsilon )\mu _k^{(2)}]\right] \ge 1-\delta /(2n) \quad \text {for all }1 \le k \le n. \end{aligned}$$

For $s\in \{1,2\}$, let $\sigma ^{(s)}_k=\sqrt{\mu ^{(s)}_k}$ be the *k*-th largest singular value of $G^{(s)}$. We can interpret $\sigma _k^{(s)}: \mathbb {C}^{n\times m} \rightarrow \mathbb {R}$ as being a function of 2*mn* i.i.d. standard (real) Gaussian random variables (the real part and imaginary part of each entry of $G^{(s)}$), and by Weyl’s inequality (Lemma 4.4), this function is 1-Lipschitz with respect to the Euclidean metric on $\mathbb {C}^{n\times m}\cong \mathbb R^{2nm}$. It follows from the Gaussian concentration inequality (Lemma 4.3) that for any $1 \le k \le n$ (and $s\in \{1,2\}$),

4.3 $$\begin{aligned} \mathbb {P}\left[ \big |\sigma _k^{(s)} - \mathbb {E}[\sigma _k^{(s)}]\big | \ge t\right] \le 2\exp (-ct^2). \end{aligned}$$

Note that our assumption $m\ge n+C\sqrt{n\log n}$, together with Lemma 4.5, yields

4.4 $$\begin{aligned} \mathbb {E}[\sigma _k^{(s)}] \ge C'\sqrt{\log n}, \end{aligned}$$

where $C^{\prime }$ can be made arbitrary large by taking large enough *C*.

We now prove (4.2). Applying (4.3) with $t = 10\sqrt{\log {(n/\delta )}}=O(\sqrt{\log n})$, and using (4.4), we see that with probability at least 1-δ/(2n), for $s\in \{1,2\}$ we have $\sigma _k^{(s)}=\mathbb E \sigma _k^{(s)}+O(\sqrt{\log n})=\mathbb E \sigma _k^{(s)}(1+O(1/C'))$. Since $\mathbb E \sigma _k^{(1)}=\mathbb E \sigma _k^{(2)}$, this implies that

$$\begin{aligned} \mu _k^{(1)}=(\sigma _k^{(1)})^2=\Big (\sigma _k^{(2)}\big (1+O(1/C')\big )\Big )^2=\mu _k^{(2)}(1+O(1/C')). \end{aligned}$$

The desired result follows, assuming $C^{\prime }$ is sufficiently large (which we may ensure by taking *C* sufficiently large in terms of ε). □

## Integral Estimates

In this section we prove the estimates in Lemma 2.5 and Lemma 2.10, via a sequence of estimates of various integrals. In this section we write f≲g to mean f=O(g).

First, for the convenience of the reader we recall the relevant parts of Definition 2.2, 2.6.

### Definition 5.1

For functions $f,g:\mathbb R\rightarrow \mathbb R$, let

$$\begin{aligned} \gamma (f)&= \frac{1}{\pi }\int _{-1}^1 f(x) \sqrt{1 - x^2}\,dx,\\ \Gamma (f,g)&=\frac{1}{\pi ^2} \int _{-1}^{1}\int _{-1}^{1} \left( \frac{f(x) - f(y)}{x-y} \right) \left( \frac{g(x) - g(y)}{x-y} \right) \frac{1 - xy}{\sqrt{1 - x^2} \sqrt{1 - y^2}}\,dx\,dy. \end{aligned}$$

Then, for $a_{\pm } = (1 \pm \sqrt{c})^2$ and $\Phi _c(x) = 2\sqrt{c} x + c + 1$, let

$$\begin{aligned} \gamma _c(f) = \frac{1}{2\pi } \int _{a_-}^{a_+} f(x)\frac{\sqrt{(a_+ - x)(x - a_-)}}{x} \,dx,\qquad \Gamma _c(f,g) = \Gamma (f\circ \Phi _c,g\circ \Phi _c). \end{aligned}$$

Now, to begin with, we show that the kernel appearing in the definition of Γ in fact yields a density of a probability measure on $[-1,1]^2$.

### Fact 5.2

We have

$$\begin{aligned} \frac{1}{\pi ^2}\int _{-1}^1 \int _{-1}^1 \frac{1 - xy}{\sqrt{(1 - x^2)(1 - y^2)}}\,dx\,dy=1. \end{aligned}$$

### Proof

With the substitution (s,t)=(1-x,1-y) and the symmetry between *s* and *t*, we compute

$$\begin{aligned}&\frac{1}{\pi ^2}\int _{-1}^1 \int _{-1}^1 \frac{1 - xy}{\sqrt{(1 - x^2)(1 - y^2)}}\,dx\,dy \\&\qquad = \frac{1}{\pi ^2}\int _0^2 \int _0^2 \frac{s + t - ts}{\sqrt{st(2 - s)(2 - t)}}\,ds\,dt \\&\qquad = \frac{2}{\pi ^2}\int _0^2 \int _0^2 \frac{\sqrt{s}}{\sqrt{t(2 - s)( 2- t)}}\,ds\,dt - \frac{1}{\pi ^2}\int _0^2 \int _0^2 \frac{\sqrt{st}}{\sqrt{(2 - s)(2 - t)}}\,ds\,dt =1, \end{aligned}$$

where we used the identities

$$\begin{aligned} \int _0^2 \sqrt{\frac{s}{2 - s}}\,ds = 2\int _0^1 \frac{x^{1/2}}{(1 - x)^{1/2}}\,dx=\pi ,\quad \int _0^2 \frac{1}{\sqrt{s(2 - s)}}\,ds = \int _0^1 \frac{x^{-1/2}}{(1 - x)^{1/2}} = \pi \end{aligned}$$

(these are Beta integrals; see for example [26, Eqns. (6.1.6) and (6.1.7)]). □

Now we proceed to study the asymptotics of the quantities appearing in Lemma 2.5, 2.10.

### Lemma 5.3

Fix $c \in [1,\infty )$, and let $h_k(x) = (x/a_+)^k.$ There is a constant $C_c \in (0,\infty )$ so that

$$\begin{aligned} \lim _{k \rightarrow \infty } k^{3/2} \gamma _c(h_k) = C_c. \end{aligned}$$

### Proof

It will be more convenient to work with $h_{k+1}$. By changing variables $x = c + 1 + (2 \sqrt{c})t$, we see

$$\begin{aligned} \gamma _c(h_{k+1})&= \frac{1}{2\pi } \int _{a_-}^{a_+} \bigg (\frac{x}{a_+}\bigg )^{k+1}\frac{\sqrt{(a_+ - x)(x - a_-)}}{x} \,dx\\&=\frac{2c}{\pi a_+} \int _{-1}^1 \left( 1 - (1 - t)\frac{2\sqrt{c}}{c + 1 + 2\sqrt{c}} \right) ^k \sqrt{(1 - t)(t + 1)}\,dt. \end{aligned}$$

Writing $\beta = \frac{2\sqrt{c}}{c + 1 + 2\sqrt{c}}$ and changing variables by 1-t=s/k, we have

$$\begin{aligned} \gamma _c(h_{k+1}) = \frac{2 c k^{-3/2}}{\pi a_+}\int _0^{2k} \left( 1 - \frac{\beta s}{k} \right) ^k \sqrt{s(2 - s/k) }\,ds. \end{aligned}$$

We note that $(1 - \beta s /k)^{k} \le e^{-\beta s}$ and so by the dominated convergence theorem, we have

$$\begin{aligned} \lim _{k \rightarrow \infty } \int _0^{2k} \left( 1 - \frac{\beta s}{k} \right) ^k \sqrt{s(2 - s/k) }\,ds = \sqrt{2} \int _0^\infty e^{-\beta s} \sqrt{s}\,ds = \frac{\sqrt{\pi }}{\sqrt{2} \beta ^{3/2}} \end{aligned}$$

(the last integral is a Gamma integral; see for example [26, Eqn. (4.1.7)]). The desired result follows. □

For the next estimate, we need the following simple inequality.

### Fact 5.4

For $1 \ge b > a \ge 0$ and k>0 we have

$$\begin{aligned} (1 - a)^k - (1 - b)^k \le e(e^{-ak} - e^{-bk}) \end{aligned}$$

### Proof

Bound

$$\begin{aligned}(1 - a)^k - (1 - b)^k = \int _a^b k (1 - \theta )^{k-1} d\theta \le \int _a^{b} k e^{-\theta (k-1)}\,d\theta \le e( e^{-ak} - e^{-bk}). \end{aligned}$$

□

### Lemma 5.5

For each fixed $c,A \in [1,\infty )$, and $h_k(x) = (x/a_+)^k$ for k≥1, we have

$$\begin{aligned} \lim _{k \rightarrow \infty } \Gamma _c(h_k,h_{Ak}) = \frac{1}{2\pi ^2} \int _0^\infty \int _0^\infty \left( \frac{e^{-s} - e^{-t}}{s - t} \right) \left( \frac{e^{-As} - e^{-At}}{s - t} \right) \frac{s + t}{\sqrt{st}} \,ds\,dt. \end{aligned}$$

### Proof

Writing $\beta = 2\sqrt{c}/a_+$ and changing variables $(x,y) = \big (1 - s/(\beta k),1 - t/(\beta k)\big )$, recalling that $\Phi _c(x)=2\sqrt{c} x+c+1$, we see that

$$\begin{aligned}&\Gamma _c(h_k,h_{Ak})\\&\quad =\frac{1}{\pi ^2} \int _{-1}^{1}\int _{-1}^{1} \left( \frac{(\Phi _c(x)/a_+)^k - (\Phi _c(y)/a_+)^k}{x-y} \right) \left( \frac{(\Phi _c(x)/a_+)^{Ak} - (\Phi _c(y)/a_+)^{Ak}}{x-y} \right) \\&\qquad \qquad \qquad \cdot \frac{1 - xy}{\sqrt{1 - x^2} \sqrt{1 - y^2}}\,dx\,dy \\&= \frac{1}{\pi ^2} \int _0^{2\beta k}\int _0^{2\beta k} \left( \frac{(1 - s/k)^k - (1 - t/k)^k}{s -t} \right) \left( \frac{(1 - s/k)^{Ak} - (1 - t/k)^{Ak}}{s -t} \right) \\&\qquad \qquad \qquad \qquad \qquad \cdot \frac{s + t - st/(\beta k)}{\sqrt{st(2 - s/(\beta k))(2 - t/(\beta k))}} \,ds\,dt. \end{aligned}$$

We may bound the integrand using Fact 5.4 by a constant multiple of

$$\begin{aligned} \left( \frac{e^{-s} - e^{-t}}{s - t} \right) \left( \frac{e^{-As} - e^{-At}}{s - t} \right) \frac{s + t}{\sqrt{st}} \end{aligned}$$

which is integrable (see Lemma 5.7, to follow), so the dominated convergence theorem completes the proof. □

### Lemma 5.6

For A≥1, we have

$$\begin{aligned} \lim _{k \rightarrow \infty } \Gamma (x^k,x^{Ak}) = \frac{1}{\pi ^2} \int _0^\infty \int _0^\infty \left( \frac{e^{-s} - e^{-t}}{s - t} \right) \left( \frac{e^{-As} - e^{-At}}{s - t} \right) \frac{s + t}{\sqrt{st}} \,ds\,dt. \end{aligned}$$

### Proof

We first claim that

5.1 $$\begin{aligned} \lim _{k \rightarrow \infty } \int _0^1 \int _{-1}^0 \left( \frac{x^k - y^k}{x - y} \right) \left( \frac{x^{Ak} - y^{Ak}}{x - y} \right) \frac{1 - xy}{\sqrt{(1 - x^2)(1 - y^2)}}\,dx\,dy = 0. \end{aligned}$$

To see this, note that on the set [0,1]×[-1,0] we may bound

$$\begin{aligned} \left( \frac{x^k - y^k}{x - y} \right) \left( \frac{x^{Ak} - y^{Ak}}{x - y} \right) \le {\left\{ \begin{array}{ll} 16 &  \text { if either } x \ge 1/2 \text { or } y \le -1/2 \\ A k^2 2^{-2(k-1)} &  \text { if } |x| \le 1/2 \text { and } |y| \le 1/2 \end{array}\right. } \end{aligned}$$

by the mean-value theorem. Applying the dominated convergence theorem shows (5.1). By symmetry of the integrand under (x,y)↦-(x,y) this shows that

$$\begin{aligned} \lim _{k \rightarrow \infty } \Gamma (x^k,x^{Ak}) \end{aligned}$$

$$\begin{aligned} = \lim _{k \rightarrow \infty } \frac{2}{\pi ^2}\int _0^1 \int _0^1 \left( \frac{x^k - y^k}{x - y} \right) \left( \frac{x^{Ak} - y^{Ak}}{x - y} \right) \frac{1 - xy}{\sqrt{(1 - x^2)(1 - y^2)}}\,dx\,dy. \end{aligned}$$

Changing variables (x,y)=(1-s/k,1-t/k) and arguing as in Lemma 5.5 completes the proof. □

### Lemma 5.7

For A≥1 we have

$$\begin{aligned} \int _0^\infty \int _0^\infty \left( \frac{e^{-s} - e^{-t}}{s - t}\right) \left( \frac{e^{-As} - e^{-At}}{s - t}\right) \frac{s+t}{\sqrt{st}}\,ds\,dt = O(A^{-1/2}(1+\log A)). \end{aligned}$$

### Proof

Since the integrand is symmetric in *s* and *t* it is sufficient to integrate over the region s<t, in which case we may bound the integrand by a constant times

$$\begin{aligned} \iint _{0 \le s < t} \left( \frac{e^{-s} - e^{-t}}{s - t}\right) \left( \frac{e^{-As} - e^{-At}}{s - t}\right) \sqrt{\frac{t}{s}}\,ds\,dt. \end{aligned}$$

We will further break up the region of integration depending on the value of t-s. For t-s≤1, bound the integrand by

$$\begin{aligned} e^{-s}\left| \frac{e^{-As} - e^{-At}}{s - t} \right| (1 + s^{-1/2}). \end{aligned}$$

(Here we used that $(e^{-s}-e^{-t})/(s-t)\le e^{-s}$, which follows from the classical inequality $1-e^{x}\le x$ with x=s-t). Then, write t=s+h, yielding

$$\begin{aligned} \iint _{0\le s \le t\le s+1} \left( \frac{e^{-s} - e^{-t}}{s - t}\right) \left( \frac{e^{-As} - e^{-At}}{s - t}\right) \sqrt{\frac{t}{s}}\,ds\,dt \end{aligned}$$

$$\begin{aligned} \le \int _0^\infty e^{-s}(1 + s^{-1/2}) e^{-As}\int _0^1 \frac{1 - e^{-Ah}}{h}\,dh \,ds. \end{aligned}$$

Now, observe that

$$\begin{aligned} \int _0^1 \frac{1 - e^{-Ah}}{h}\,dh&= \int _0^{1/A} \frac{1 - e^{-Ah}}{h}\,dh + \int _{1/A}^1 \frac{1 - e^{-Ah}}{h}\,dh \\&\le \int _0^{1/A} A \,dh + \int _{1/A}^1 h^{-1}\,dh \\&= 1 + \log A, \end{aligned}$$

and

5.2 $$\begin{aligned} \int _0^\infty e^{-s}(1 + s^{-1/2}) e^{-As}&\le \frac{1}{\sqrt{A}}+\sqrt{A+1}\int _{1/\sqrt{A}}^\infty ((A+1)s)^{-1/2} e^{-(A+1)s}\nonumber \\&=\frac{1}{\sqrt{A}}+\frac{1}{\sqrt{A+1}}\int _{1}^\infty q^{-1/2} e^{-q}\lesssim \frac{1}{\sqrt{A}}. \end{aligned}$$

We deduce that

$$\begin{aligned} \iint _{0\le s \le t\le s+1} \left( \frac{e^{-s} - e^{-t}}{s - t}\right) \left( \frac{e^{-As} - e^{-At}}{s - t}\right) \sqrt{\frac{t}{s}}\,ds\,dt \lesssim \frac{1+\log A}{\sqrt{A}}. \end{aligned}$$

This takes care of the region where t-s≤1; we now turn to the complementary region t-s≥1. With the substitution q=(t-s)/s we first compute

$$\begin{aligned} \int _{s + 1}^\infty (t-s)^{-2} \sqrt{1 + \frac{(t-s)}{s}}dt=s\int _{1/s}^\infty q^{-2}\sqrt{1+q}\,dq\lesssim s^{-1/2}. \end{aligned}$$

Combining this with (5.2), we have

$$\begin{aligned}&\iint _{t \ge s + 1} \left( \frac{e^{-s} - e^{-t}}{s - t}\right) \left( \frac{e^{-As} - e^{-At}}{s - t}\right) \sqrt{\frac{t}{s}}\,ds\,dt \\&\le \int _0^\infty e^{-s(1 + A)} \int _{s + 1}^\infty (t-s)^{-2} \sqrt{1 + \frac{(t-s)}{s}} \,dt\,ds \\&\lesssim \int _0^\infty e^{-s(1 + A)} s^{-1/2} \,ds\lesssim \frac{1}{\sqrt{A}}. \end{aligned}$$

□

### Proof of Lemma 2.5

Note that (1) follows from Lemma 5.3. The other two items follow by combining Lemma 5.5 with Lemma 5.7. □

### Proof of Lemma 2.10

Both items follow by combining Lemma 5.6 with the estimate from Lemma 5.7. □

## Data Availability

No datasets were used in this research.

## A General Central Limit Theorem for Linear Statistics of the Wishart–Laguerre Ensemble

In this appendix we explain how to deduce the statements of Theorem 2.3, 2.7 from results in [5, 6, 23]. As in the statements of these theorems, let $\mu _1,\dots ,\mu _n$ be the eigenvalues of a random matrix sampled from the complex Wishart–Laguerre ensemble with parameters *n*, *m*, and assume $m/n\rightarrow c\in [1,\infty ]$.

We recall the relevant statements from [5, 6, 23]. Let $$a_\pm = (1\pm \sqrt{c})^2$$, and let $$\mathcal {F}_0$$ be the set of all differentiable functions $$f:\mathbb R\rightarrow \mathbb R$$ such that both *f* and $f^{\prime }$ have compact support. The cited results are stated under slightly different regularity assumptions, but $$\mathcal {F}_0$$ is a convenient common subclass which certainly satisfies each of them. Below we will use truncation and concentration to pass from $$\mathcal {F}_0$$ to the larger class F appearing in Theorem 2.3, 2.7; in particular, the polynomial test functions used in Corollary 2.4 and 2.8 belong to F. Fix $$g\in \mathcal F_0$$.

First, if c<∞ then let $W=g(\mu _1/n)+\dots +g(\mu _n/n)$. In this case, [23, Theorem 4.1] (which is really a restatement of results in [24]) says that

A.1 $$\begin{aligned} \frac{W}{n}\overset{p}{\rightarrow }\frac{1}{2\pi }\int _{a_-}^{a_+} g(\lambda )\frac{\sqrt{(\lambda -a_-)(a_+-\lambda )}}{\lambda }d\lambda = \gamma _c(g),\end{aligned}$$

and [23, Theorem 4.2] says that $W-\mathbb EW\overset{d}{\rightarrow }\mathcal N(0,\sigma ^2)$, where

A.2 $$\begin{aligned} \sigma ^2&=\frac{1}{2}\cdot \frac{1}{2\pi ^2}\int _{a_-}^{a_+}\int _{a_-}^{a_+}\left( \frac{g(\lambda _1) - g(\lambda _2)}{\lambda _1 - \lambda _2}\right) ^2 \frac{4c - (\lambda _1 - c - 1)(\lambda _2 - c - 1)}{\sqrt{4c - (\lambda _1 - c - 1)^2}\sqrt{4c - (\lambda _2 - c - 1)^2}}\,d\lambda _1d\lambda _2 \nonumber \\&= \Gamma _c(g,g). \end{aligned}$$

Second, if c=∞, then with $X=g((\mu _1-m)/2\sqrt{mn})+\dots +g((\mu _n - m)/2\sqrt{mn})$, the convergence to the semicircle law proved in [5] implies that

A.3 $$\begin{aligned} \frac{X}{n}\overset{p}{\rightarrow }\frac{2}{\pi }\int _{-1}^{1}g(\lambda )\sqrt{1-\lambda ^2}d\lambda = \gamma (g).\end{aligned}$$

With the slightly different parameterisation $Y=g((\mu _1-m)/\sqrt{mn})+\dots +g((\mu _n - m)/\sqrt{mn})$, [6, Theorem 1.2] says that $Y-\mathbb EY\overset{d}{\rightarrow }\mathcal N(0,\sigma ^2)$, where

A.4 $$\begin{aligned} \sigma ^2=\frac{1}{2}\cdot \frac{1}{2\pi ^2}\int _{-2}^{2}\int _{-2}^{2}\left( \frac{g(\lambda _1) - g(\lambda _2)}{\lambda _1 - \lambda _2}\right) ^2 \frac{4 - \lambda _1\lambda _2}{\sqrt{4 - \lambda _1^2}\sqrt{4 - \lambda _2^2}}\,d\lambda _1d\lambda _2 = \Gamma (g,g).\end{aligned}$$

### Remark

The above cited papers all primarily concern the *real* Wishart–Laguerre ensemble; we need the complex case. The same proofs apply to both cases (the only difference is that the variance in the real case is a factor of 2 larger than the variance in the complex case). See [23, Remark 4.1].

These results imply the univariate cases of Theorem 2.3, 2.7, assuming that $f \in \mathcal {F}_0$ and provided we weaken $L^1$-convergence to convergence in probability. We now discuss how to upgrade this to the desired statements, using known results on concentration of singular values of Gaussian random matrices. Specifically, we will need the following.

### Lemma A.1

Let $\mu _1 \ge \dots \ge \mu _n$ be the eigenvalues of a random matrix sampled from the complex Wishart–Laguerre ensemble with parameters m≥n. There exists an absolute constant *C* such that for any t≥0,

$$\begin{aligned} \sqrt{m} - C(\sqrt{n}+t)\le \sqrt{\mu _n} \le \sqrt{\mu _1} \le \sqrt{m} + C(\sqrt{n}+t) \end{aligned}$$

with probability at least $1 - 2\exp (-t^2)$.

### Remark

In the real case, this statement is proved in [39, Theorem 4.6.1], but the same proof extends readily to the complex case as well.

Therefore, in the case c<∞, it follows from the upper bound in Lemma A.1 that for all sufficiently large *s* (depending on *c*),

$$\begin{aligned} \mathbb {P}\Big [\max _{k} \mu _k/n \ge s\Big ] = \mathbb {P}[\sqrt{\mu _1} \ge \sqrt{sn}] \le \mathbb {P}[\sqrt{\mu _1} \ge \sqrt{m} + C\sqrt{n} + C\sqrt{sn/2}] \le 2\exp (-sn/4), \end{aligned}$$

so that if $g \in \mathcal {F}$ (i.e. *g* is continuously differentiable and has sub-exponential growth) then for any ε>0, we can (smoothly) truncate $g \in \mathcal {F}$ to some function $g_0 \in \mathcal {F}_0$ with compact support, in such a way that

$$\begin{aligned} \mathbb E\Big |\Big (g\big (\mu _1/n)\big )+\dots +g\big (\mu _n/n)\big )\Big )-\Big (g_0\big (\mu _1/n\big )+\dots +g_0\big (\mu _n/n\big )\Big )\Big |\le \varepsilon . \end{aligned}$$

That is to say, the effect of the truncation can be made arbitrarily small, so if the asymptotic results in (A.1),(A.2) hold for every $g\in \mathcal F_0$ they must also hold for every $g\in \mathcal F$.

Further, by the upper bound in Lemma A.1 and using that $g \in \mathcal {F}$ has sub-exponential growth, it follows that the sequence of random variables *W*/*n* is uniformly integrable. Since this sequence converges to $\gamma _c(g)$ in probability by (A.1), it follows by uniform integrability that it also converges in $L^1$.

In the case c=∞, we use both the upper and the lower bound in Lemma A.1 to see that for all sufficiently large *s*,

$$\begin{aligned} \mathbb {P}\Big [\max _k (\mu _k - m)/\sqrt{mn} \ge s\Big ]&\le \mathbb {P}[\mu _1 \ge m + s\sqrt{mn}] + \mathbb {P}[\mu _n \le m - s\sqrt{mn}]\\&\le 2\min \{\exp (-s^2 n/2), \exp (-s\sqrt{mn}/2)\}\\&\le 2\exp (-sn/4), \end{aligned}$$

from which it follows (as before) that we can upgrade the assumption $g \in \mathcal {F}_0$ to the assumption $g \in \mathcal {F}$ and also upgrade the convergence in (A.3) to convergence in $L^1$.

Finally, we discuss how to deduce multivariate central limit theorems from the above univariate central limit theorems. Given functions $f_1,\dots ,f_\ell \in \mathcal F$ and a vector $\textbf{t}=(t_1,\dots ,t_\ell )\in \mathbb R^\ell$, define

$$ f_{\textbf{t}}=t_1f_1+\dots +t_\ell f_\ell . $$

Applying the above univariate convergence to the function $f_{\textbf{t}}$, we obtain in the case c<∞ that

$$ t_1(W_1-\mathbb E W_1)+\dots +t_\ell (W_\ell -\mathbb E W_\ell ) \overset{d}{\rightarrow }\mathcal N\bigl (0,\Gamma _c(f_{\textbf{t}},f_{\textbf{t}})\bigr ), $$

and in the case c=∞ that

$$ t_1(Y_1-\mathbb E Y_1)+\dots +t_\ell (Y_\ell -\mathbb E Y_\ell ) \overset{d}{\rightarrow }\mathcal N\bigl (0,\Gamma (f_{\textbf{t}},f_{\textbf{t}})\bigr ). $$

By bilinearity,

$$ \Gamma _c(f_{\textbf{t}},f_{\textbf{t}})=\sum _{a,b=1}^\ell t_a t_b\,\Gamma _c(f_a,f_b), \qquad \Gamma (f_{\textbf{t}},f_{\textbf{t}})=\sum _{a,b=1}^\ell t_a t_b\,\Gamma (f_a,f_b). $$

Thus the covariance forms appearing here are exactly those associated with the matrices

$$ \Sigma _c=\bigl (\Gamma _c(f_a,f_b)\bigr )_{a,b\le \ell }, \qquad \Sigma _\infty =\bigl (\Gamma (f_a,f_b)\bigr )_{a,b\le \ell }. $$

The desired multivariate convergences in Theorem 2.3, 2.7 now follow from the Cramér–Wold device; see, for example, [8, Theorem 29.4].

## Footnotes

1 We could equally well have defined these eigenvalues by tracing out Alice’s subsystem, instead of Bob’s subsystem. It is not hard to see that this choice only affects the number of zero eigenvalues; the nonzero eigenvalues do not actually depend on which of the two subsystems we choose to trace out.

2 Note that since the eigenvalues sum to 1, this is equivalent to the condition that the sum of the *k* *largest* eigenvalues of $\rho _\psi$ is at most the sum of the *k* *largest* eigenvalues of $\rho _\phi$, for all k≤n.

3 Random pure states have been studied for many different purposes throughout the physics literature (with varying levels of rigour), and it is a bit difficult to definitively pin down the origins of this observation. The distribution of the entanglement spectrum of a random bipartite state (which has now become known as the *Hilbert–Schmidt measure*) was perhaps first studied by Lubkin [21], and an explicit formula for the density function was first computed by Lloyd and Pagels [20]. The connection to the Wishart–Laguerre spectrum seems to have been first explicitly observed by Życzkowski and Sommers [43].

4 There are two slightly different conventions for the definition of “complex standard Gaussian” (differing by a factor of $\sqrt{2}$). Due to the trace-normalisation, the distinction is irrelevant, but for concreteness we use the normalisation that for complex standard Gaussian *X* we have $\mathbb E[(\Re X)^2+(\Im X)^2]=1$.
